# Anti-Phosphatidylserine/Prothrombin Antibodies at Two Points: Correlation With Lupus Anticoagulant and Thrombotic Risk

**DOI:** 10.3389/fimmu.2021.754469

**Published:** 2021-10-21

**Authors:** Natalia Egri, Chelsea Bentow, Laura Rubio, Gary L. Norman, Susana López-Sañudo, Michael Mahler, Albert Pérez-Isidro, Ricard Cervera, Odette Viñas, Gerard Espinosa, Estíbaliz Ruiz-Ortiz

**Affiliations:** ^1^ Institut de Recerca Biomèdica August Pi i Sunyer (IDIBAPS), Barcelona, Spain; ^2^ Department of Immunology, Centre de Diagnòstic Biomèdic, Hospital Clínic de Barcelona, Barcelona, Spain; ^3^ Headquarters & Technology Center, Autoimmunity, Werfen, San Diego, CA, United States; ^4^ Autoimmunity Line, Werfen, Madrid, Spain; ^5^ Department of Autoimmune Diseases, Hospital Clínic de Barcelona, Barcelona, Spain

**Keywords:** anti-phospholipid syndrome, anti-phosphatidylserine/prothrombin antibodies, thrombosis, pregnancy morbidity, anti-phospholipid antibodies

## Abstract

Antibodies to phospholipids (aPL) and associated proteins are a hallmark in the diagnosis of anti-phospholipid syndrome (APS). Those included in the classification criteria are the lupus anticoagulant (LA) and the IgG and IgM isotypes of anticardiolipin (aCL) and anti-beta-2 glycoprotein I (β2GPI) antibodies. Non-classification criteria markers such as autoantibodies that recognize the phosphatidylserine/prothrombin (aPS/PT) complex have been proposed as biomarkers for APS. Studies of aPS/PT antibodies have shown a strong correlation to clinical manifestations and LA. We aimed to study the value and the persistence of aPS/PT IgG and IgM antibodies in a cohort of consecutive patients with clinical suspicion of APS and their utility as thrombotic risk markers. Our study, with 103 patients, demonstrates that persistently positive results for aPS/PT IgG antibodies were significantly associated with APS classification, thrombosis, triple aPL positivity, LA positive result, and the Global APS Score (GAPSS) > than 9 points (p < 0.01, for each condition). On the other hand, no association was seen with pregnancy morbidity (p = 0.56) and SLE (p = 0.07). Persistence of aPS/PT antibodies, defined according to the current laboratory classification criteria, likely improves the diagnosis and clinical assessment of patients with APS.

## Introduction

Antiphospholipid syndrome (APS) is a systemic autoimmune disease that is characterized by vascular thrombosis and/or well-defined obstetric complications that occur in patients with persistent anti-phospholipid (aPL) antibodies (Ab) (1). The aPL Ab included in the current laboratory criteria are anti-cardiolipin (aCL) and anti-β2-glycoprotein I (aβ2GPI) Ab of either IgG or IgM isotype and lupus anticoagulant (LA) (2). Non-classification criteria markers, such as Ab that recognize other phospholipid (PL) or PL-associated proteins like the phosphatidylserine/prothrombin (PS/PT) complex, have been proposed as biomarkers for seronegative APS patients (3).

It seems clear that in APS Ab profiles, rather than isolated results, best define the risk of patients to develop the clinical manifestations of this syndrome (4). In this sense, including new Ab can add value to improve the stratification of patients and help in the interpretation of results, since discrepant results often appear for different reasons. For example, LA cannot be determined in the presence of classic anticoagulant treatments, heparin, and vitamin K antagonists (VKAs), due to the presence of false-positive results (5). Guidelines recommend performing laboratory procedures after low molecular weight heparin has been discontinued for at least 12 h or, in the case of VKAs, 2 weeks after discontinuation or until an international normalized ratio (INR) of ≤1.5 has been achieved (6). Various studies have been conducted to assess whether this effect also appeared with the use of new direct oral anticoagulants (DOACs) that directly inhibit a specific factor in the coagulation cascade, for example, those targeted to thrombin and factor Xa, which are used worldwide to prevent and to treat thromboembolism, embolic stroke associated with non-valvular atrial fibrillation, and acute coronary syndromes (7). Depending on the test used for LA determination, based on different principles, in patients treated with DOACs, different results were obtained. At this time, it does not seem advisable to carry out LA testing during anti-factor Xa and anti-factor IIa treatment because of the risk of false-positive results (8). It is recommended to wait at least 72 h after the last dose of DOACs for the investigation of LA (9).

Numerous studies have shown a close association between the presence of LA and aPS/PT Ab in patients with APS, with aPS/PT acting as a potential surrogate LA confirmatory test but independent of LA presence (10). LA has also been found to be an independent risk factor for thrombosis in aPL carriers (11). These findings have been confirmed by a recent meta-analysis showing that LA is associated with a higher risk for thrombotic events with respect to aCL and aβ2GPI Ab (12). In this sense, aPS/PT Ab strongly correlate with thromboembolic events (13). While to date, aPS/PT Ab are not included in the APS laboratory criteria, their positivity has been recently proposed as a part of both the Global APS Score (GAPSS) (14) and the aPL Score (aPL-S) (15). Furthermore, in a study of 23 different combinations of aPL antibodies in a SLE cohort, it was demonstrated that the best diagnostic accuracy and the highest risk for thrombosis and pregnancy loss corresponded to the combination of LA, aβ2GPI, and aPS/PT instead of the current laboratory classification criteria (16). In addition, false-positive results for aPS/PT Ab have not been demonstrated when the determination is made in patients who are receiving anticoagulant treatment (17). For this reason, these Ab could be a substitute marker for LA in patients on anticoagulant therapy (9, 18) in the future as well as add value for the stratification of APS patients. Due to the long-standing difficulties in standardization of aPL Ab detection assays, an evaluation of the diagnostic performance of novel technologies is still needed.

The aim of the present study was to investigate the value that IgG and IgM aPS/PT Ab, which are not included in the current APS classification criteria, can add to APS diagnosis as well as their possible role as thrombotic risk markers in a cohort of patients with APS and to evaluate the persistence of these Ab in the same cohort of patients.

## Material and Methods

### Patient Population

A total of 103 patients from the Hospital Clínic of Barcelona referred for aPL Ab testing between 2016 and 2018 were retrospectively randomly selected and included in the study.

Four study groups were included: 25 (24%) patients with APS, 17 of them with primary APS; 30 (29%) patients with systemic lupus erythematous (SLE); 22 (21%) patients defined as non-APS, but who suffered from thrombosis and/or obstetric complications included in the classification criteria for APS (19) in the absence of positive aPL in two determinations (n = 19) or with only one positive aPL determination (n = 3); and 26 (25%) patients who were referred for aPL testing for other reasons (Others group). The latter was a heterogeneous group of patients with various autoimmune diseases (n = 17), subclinical hypothyroidism (n = 3), and type 2 diabetes mellitus (n = 2), and four patients each presenting with aortic aneurysm, arthralgia, ischemic cardiomyopathy, and uveitis and skin lesions, respectively. Classification of APS was determined using Sydney criteria (19). All SLE patients fulfilled the 2019 European League Against Rheumatism/American College of Rheumatology Classification Criteria for SLE classification criteria (20).

All participants had given written informed consent and the inclusions were performed in agreement with Declaration of Helsinki. Approval was obtained from Ethical Committee of Hospital Clínic Barcelona (HCB/2019/1046).

### Methods

Serum samples from two different blood draws separated by at least 12 weeks were collected from all patients.

The study of aCL and aβ2GPI IgG and IgM was performed by chemiluminescence assay (CIA) (QUANTA Flash^®^, Inova Diagnostics, CA). The cutoff recommended by the manufacturer is 20 CU. aPS/PT IgG and IgM determination were performed by ELISA (QUANTA Lite^®^, Inova Diagnostics, CA). The cutoff recommended by the manufacturer is 30 U/ml. LA was determined according to the International Society of Thrombosis and Hemostasis-Scientific Standardization Subcommittee (ISTH-SSC) guideline (6).

Triple aPL positivity was considered when the patient had a positive result for aCL and aβ2GPI of IgG/IgM isotype in addition to LA. GAPSS was calculated, taking into account arterial hypertension, hyperlipidemia, and LA, aCL, aβ2GPI, and aPS/PT results.

### Statistical Analysis

The prevalence of Ab was measured based on the manufacturer’s recommended cutoffs. Descriptive statistics were presented as mean or median for continuous variables and number or percentage for categorical variables. Wilcoxon Mann–Whitney and ANOVA tests were used to compare continuous variables, and Fisher’s exact test was used to compare categorical variables. p values <0.05 were considered statistically significant. Analyse-it^®^ for Excel method evaluation software (version 5.40.2; Leeds, UK) was used for statistical analyses.

## Results

### Clinical Characteristics and Results of Classical APS Biomarkers

The patient population consisted of 86 (83.5%) women and 17 (16.5%) men. The median age at data collection was 46 years. Patients’ demographics and clinical characteristics by disease group are summarized in **Table 1**. The distribution and prevalence of the classical APS markers (aCL and aβ2GPI Ab and LA) in our cohort are also included. Thrombosis was present in 34 (33%) patients and pregnancy morbidity in 16 out of 28 (57.1%) women with pregnancies.

**Table 1:** Demographic characteristics, clinical manifestations, and APS laboratory features of patients included according to each group.

	APS	Non-APS	SLE	Others
	n = 25	n = 22	n = 30	n = 26
**Demographic characteristics**				
Age (median 95% CI)	52 (44–57)	48 (40–64)	47 (40–53)	53 (38–69)
Female gender	19 (76)	18 (82)	29 (97)	20 (77)
**APS type:**				
Primary APS	17 (68)	–	–	–
Associated APS with SLE	8 (32)	–	–	–
**APS clinical manifestations**				
Thrombosis	19 (76)	9 (41)	0 (0)	1 (4)
Pregnancy morbidity	5 (20)	9 (41)	1 (3)	1 (4)
Both thrombosis and pregnancy morbidity	1 (4)	4 (18)	0 (0)	0 (0)
Thrombosis:	Total n = 20	Total n = 13	Total n = 0	Total n = 1
• Arterial/venous	13/6/1	5/7/1		0/1
Pregnancy morbidity:	Total n = 5	Total n = 10	Total n = 0	Total n = 1
• Early/late/premature delivery	2/3/0	7/2/1	0/0/0	1/0/0
**aPL testing** (first determination)				
LA	19 (76)	2 (9)	3 (10)	3 (11)
aCL Ab IgG	16 (64)	1 (5)	6 (20)	0 (0)
aCL Ab IgM	10 (25)	0 (0)	4 (13)	6 (23)
aβ2GPI Ab IgG	19 (76)	0 (0)	8 (27)	3 (11)
aβ2GPI Ab IgM	8 (32)	0 (0)	5 (17)	3 (11)
**APS laboratory criteria** (aPL positive in two different blood draw separated for at least 12 weeks)				
LA *^a^*	7/12 (58)	0/11 (0)	1/5 (20)	1/8 (12)
aCL Ab IgG	16 (64)	0 (0)	5 (17)	0 (0)
aCL Ab IgM	10 (25)	0 (0)	4 (13)	6 (23)
aβ2GPI Ab IgG	19 (76)	0 (0)	8 (27)	2 (8)
aβ2GPI Ab IgM	8 (32)	0 (0)	5 (17)	3 (11)
**Risk factors for thrombosis**				
Triple aPL positivity	15 (60)	0 (0)	3 (10)	2 (8)
Arterial hypertension	9 (36)	4 (19)	6 (20)	8 (33)
Hyperlipidemia^*b*^	8 (32)	6 (40)	10 (17)	6 (27)
GAPSS ≥ 9^*c*^	21 (84)	0 (0)	7 (29)	4 (19)
**Anticoagulation treatment**				
Anticoagulated at sampling^*d*^	18 (72)	8 (53)	2 (8)	0 (0)

Values of categorical variables are expressed as number and (percentage).

a Second testing only performed in n = 36 patients (results expressed as +ve/analyzed).

b Data available for 86 patients.

c Calculated for 85 patients.

d Data available for 85 patients.

APS, anti-phospholipid syndrome; SLE, systemic lupus erythematous; aPL, anti-phospholipids; LA, lupus anticoagulant; aCL Ab, anti-cardiolipin antibodies; aβ2GPI Ab, anti-β2-glycoprotein I antibodies; GAPSS, Global Antiphospholipid Syndrome Score.

All patients included in this work were evaluated for classical Ab at two temporal points separated by at least 12 weeks. The results obtained in each group of patients for the aPL Ab included in the laboratory criteria (IgG/IgM aCL and aβ2GPI) are shown in **Table 1**. Twenty out of 103 (19.4%) patients were triple positive, with 60% (15/20) from the APS group.

### aPS/PT Antibody Results

As in the case of classical biomarkers, patients were also evaluated for aPS/PT Ab at two points, separated by at least 12 weeks. Considering aPS/PT Ab, in the first serum sample, we identified aPS/PT IgG Ab positivity in 32% of APS patients, 5% in non-APS, 13% in SLE, and 8% in Others, respectively (**Table 2**). In the case of the IgM isotype, in first sample, we found a positive result in 44% (45/103) of patients tested and 40% (18/45) in APS patients (**Table 2**). Similar findings (non-fluctuating aPS/PT Ab results) were seen with second serum samples from a follow-up blood draw separated by >12 weeks. Persistent positivity rates for aPS/PT IgG and IgM Ab were 28% and 72% for APS, 0% and 18% for non-APS, 7% and 30% for SLE, and 0% and around 30% for Others, respectively. Prevalence of aPS/PT IgG and IgM antibodies compared to LA, aCL, and aβ2GPI at first sample were also analyzed (**Table 3**). The best agreement was obtained between the aPS/PT IgG Ab and LA (Cohen kappa: 0.84). Due to the better results obtained for IgG aPS/PT Ab (specificity 84%) versus IgM (specificity 50%), we decided to more extensively analyze the IgG isotype. Overall, 19 (18.4%) patients were positive for aPS/PT IgG Ab, at either one (n = 10) or both (n = 9) sample points. Patients with at least one sample positive for aPS/PT IgG were 8/19 APS, 2/19 non-APS, 5/19 SLE, and 4/19 Others. The correlation of aPS/PT IgG Ab levels between the first and second samples for those 19 patients was calculated and showed a Spearman’s ratio of 0.72 (95% CI 0.38–0.89).

**Table 2:** Prevalence of aPS/PT IgG and IgM antibodies by disease group.

	Total	APS	Non-APS	SLE	Others
	n = 103	n = 25	n = 22	n = 30	n = 26
**aPS/PT IgG**					
First sample	15 (15)	8 (32)	1 (5)	4 (13)	2 (8)
Second sample	13 (13)	7 (28)	1 (5)	3 (10)	2 (8)
Both	9 (9)	7 (28)	0 (0)	2 (7)	0 (0)
At least one positive	19/103 (18)	8/25 (32)	2/22 (9)	5/30 (17)	4/26 (15)
**aPS/PT IgM**					
First sample	45 (44)	18 (72)	4 (18)	13 (43)	10 (38)
Second sample	45 (44)	19 (76)	5 (22)	12 (40)	9 (35)
Both	38 (37)	18 (72)	4 (18)	9 (30)	7 (27)
At least one positive	52/103 (51)	19/25 (76)	5/22 (23)	16/30 (53)	12/26 (46)
aPS/PT IgM+ IgG- at first sample	31/103 (30)	10/25 (40)	4/22 (18)	9/30 (30)	8/26 (31)

Values of categorical variables are expressed as number and (percentage). Second samples were collected > 12 weeks apart.

APS, anti-phospholipid syndrome; SLE, systemic lupus erythematous; aPS/PT, anti-phosphatidylserine/prothrombin antibodies.

**Table 3:** Prevalence of aPS/PT IgG and IgM antibodies compared to LA, aCL, and aβ2GPI at first sample.

	APS/SLE/others patients (n = 79)									
	LA		aCL Ab IgG		aβ2GPI Ab IgG		LA-aCL-aβ2GPI Ab IgG		aCL-aβ2GPI Ab IgG	
	Positive	Negative	Positive	Negative	Positive	Negative	Triple positive	Triple negative	Double positive	Double negative
	n = 25	n = 54	n = 22	n = 57	n = 30	n = 49	n = 16	n = 44	n = 5	n = 44
aPS/PT Ab IgG	Kappa: 0.84		Kappa: 0.36		Kappa: 0.22		Kappa: 0.38		Kappa: 0.11 (0 ∈ 95%IC)	
Positive	25 (100)	6 (11)	9 (41)	5 (9)	9 (30)	5 (10)	7 (44)	4 (9)	1 (20)	4 (9)
Negative	0 (0)	48 (89)	13 (59)	52 (91)	21 (70)	44 (90)	9 (56)	40 (91)	4 (80)	40 (91)
	**LA**		**aCL Ab IgM**		**aβ2GPI Ab IgM**		**LA-aCL-aβ2GPI Ab IgM**		**aCL-aβ2GPI Ab IgM**	
	**Positive**	**Negative**	**Positive**	**Negative**	**Positive**	**Negative**	**Triple positive**	**Triple negative**	**Double positive**	**Double negative**
	**n = 25**	**n = 54**	**n = 20**	**n = 59**	**n = 16**	**n = 63**	**n = 7**	**n = 42**	**n = 5**	**n = 42**
aPS/PT Ab IgM	Kappa: 0.35		Kappa: 0.33		Kappa: 0.23		Kappa: 0.42		Kappa: 0.26	
Positive	20 (80)	21 (39)	17 (85)	24 (41)	13 (81)	28 (44)	7 (100)	12 (29)	4 (80)	12 (29)
Negative	5 (20)	33 (61)	3 (15)	35 (59)	3 (19)	35 (56)	0 (0)	30 (71)	1 (20)	30 (71)

Values of categorical variables are expressed as number and (percentage). Cohen’s kappa interpretation: poor <0.20; weak 0.21–0.40; moderate 0.41–0.60; good 0.61–0.80; very good 0.81–1.00.

APS, anti-phospholipid syndrome; SLE, systemic lupus erythematous; aPS/PT, anti-phosphatidylserine/prothrombin antibodies.

**Figure 1:**
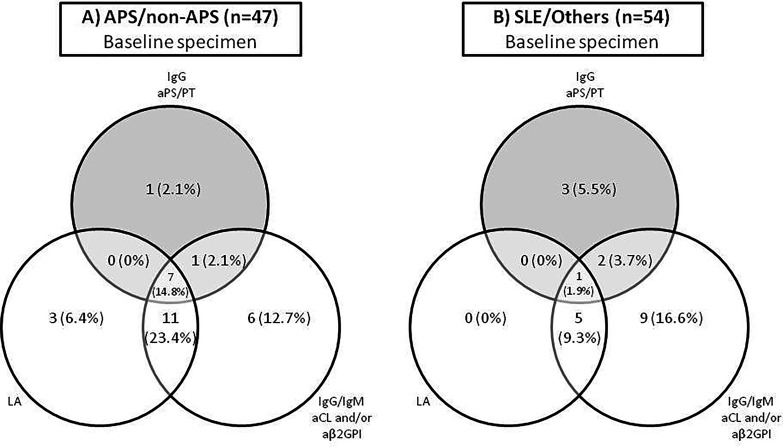
The distribution of aPL biomarker reactivity in **(A)** APS/non-APS samples and **(B)** SLE/Others samples.

Positivity for aPS/PT IgG Ab was significantly associated with APS classification by criteria, thrombosis, and other clinical parameters related to higher risk of thrombosis such as triple aPL positivity and GAPSS > 9 points. Positivity for aPS/PT IgG Ab was also significantly associated with LA positive result. No association was seen with pregnancy morbidity and SLE. The odds ratio increases when both determinations are positive (**Table 4**). In relation to those patients with clinical manifestations of APS (APS and non-APS groups), seven patients were triple positive (LA, IgG/IgM aCL, and/or aβ2GPI, as well as IgG aPS/PT) versus only one triple-positive patient in the SLE/Others group (**Figure 1**). Similarly, significant differences were observed between aPS/PT IgG Ab levels, taking into account average values between first and second determination, and these same clinical associations (**Table 5**).

**Table 4:** Association between aPS/PT IgG antibody positivity rate and clinical manifestations.

Clinical parameter	Total of 103 patients n (%)	At least one positive determination *vs*. double negatives				Both determinations with positive results *vs*. negative or single positive			
		aPS/PT IgG Pos (n = 15)	aPS/PT IgG Neg (n = 88)	*p value*	Odds ratio (95% CI)	aPS/PT IgG Pos (n = 9)	aPS/PT IgG Neg (n = 94)	*p value*	Odds ratio (95% CI)
APS	25 (24)	8 (53)	17 (19)	**<0.01**	4.7 (1.3–17.6)	7 (78)	18 (19)	**<0.01**	14.3 (2.5–151.8)
Thrombosis	34 (33)	9 (60)	25 (28)	**<0.05**	3.7 (1.1–14.2)	7 (78)	27 (29)	**<0.01**	8.5 (1.5–88.8)
Pregnancy morbidity*^a^*	16/28 (56.1)	1 (20)	15 (65)	0.1331	0.144 (0.0003–1.770)	1 (33)	15 (60)	0.5604	0.347 (0.005–7.503)
SLE	38 (37)	9 (60)	29 (33)	0.08	3.0 (0.9–11.4)	2 (22)	32 (34)	0.07	3.8 (0.8–25.2)
Triple Positive	20 (19)	7 (47)	13 (15)	**<0.01**	5.0 (1.3–18.9)	7 (78)	13 (14)	**<0.01**	20.8 (3.5–225.8)
LA	27/101 (27)	8 (53)	19/86 (22)	**<0.05**	4.0 (1.1–14.7)	8 (89)	19/86 (22)	**<0.01**	29.5 (3.6–1381.6)
GAPSS > 9	32/85 (38)	10/14 (71)	22/71 (31)	**<0.01**	5.4 (1.4–26.5)	8/9 (89)	24/76 (32)	**<0.01**	16.8 (2.1–783.4)

Values of categorical variables are expressed as number and (percentage).

^a^Data calculated on the total number of women who have had at least one pregnancy (n = 28).

APS, anti-phospholipid syndrome; SLE, systemic lupus erythematous; LA, lupus anticoagulant; GAPSS, Global Antiphospholipid Syndrome Score; Pos, positive; Neg, negative; aPS/PT, anti-phosphatidylserine/prothrombin antibodies. Bold means statistically significant values.

**Table 5:** Association between aPS/PT IgG antibody levels and APS clinical manifestations.

Clinical parameter	Total = 103 n (%)	Median (CI 95%) if present	Median (CI 95%) if absent	*p*	Hodges–Lehmann shift (95% CI)
APS	25 (24)	11.5 (8.9–27.0)	9.0 (7.7–11.6)	**<0.01**	**3.5 (1.36–9.06)**
Thrombosis	34 (33)	12.1 (8.4–20.3)	9.2 (7.6–11.6)	**0.01**	**2.7 (0.67–7.37)**
Pregnancy morbidity*^a^*	16 (57)	9.0 (7.6–15.0)	13.4 (5.8–28.7)	0.43	-2.8 (-17.4–2.63)
SLE	38 (37)	12.3 (9.2–15.7)	8.7 (8.0–10.2)	0.06	2.5 (-0.11–5.34)
Triple positive	20 (19)	15.8 (10.2–96.3)	8.8 (7.9–11.1)	**<0.01**	**6.3 (2.5–17.1)**
LA	27/101 (27)	12.3 (8.8–88.5)	9.2 (7.7–11.5)	**<0.01**	**3.2 (1.2–8.7)**
GAPSS > 9	32/85 (38)	3.3 (9.2 – 27.0)	8.7 (7.6 – 11.3)	**<0.01**	**4.6 (1.4–9.0)**

^a^Data calculated on the total number of women who have had at least one pregnancy (n = 28).

APS, anti-phospholipid syndrome; SLE, systemic lupus erythematous; LA, lupus anticoagulant; GAPSS, Global Antiphospholipid Syndrome Score. Bold means statistically significant values.

This study confirms the need to include the detection of other Ab, such as those against the PS/PT complex, in the diagnostic criteria for APS. As with classical Ab, the determination of aPS/PT Ab must be carried out at two points.

## Discussion

This study aimed to investigate the value of IgG and IgM aPS/PT Ab by ELISA, analyzing the persistence of these Ab and its possible role as a thrombotic risk marker in a cohort of patients with APS. aPS/PT Ab have been included in the GAPSS (14) and the aPL-Score (Otomo) but, to date, are not considered in the current classification criteria. Our study clearly demonstrated a higher prevalence of IgG/IgM aPS/PT in the APS group compared with the non-APS and other autoimmune disease groups. In our cohort, the prevalence for aPS/PT IgG Ab in the APS and non-APS groups was 32% and 9%, respectively. This prevalence was in line with previous findings. Litvinova et al. (21) detected the presence of IgG aPS/PT in 43.9% of APS patients and in 5.6% of seronegative APS. On the contrary, Pregnolato et al. (22) detected higher prevalence (81.3%) for APS diagnosis due to the fact that they took into account both IgG and IgM isotypes, but in the case of only IgG aPS/PT, the prevalence decreased to 40%. As recommended in the current classification APS criteria for the classic aPL, we have carried out a second determination of aPS/PT Ab (IgG and IgM) at least 12 weeks after the first evaluation. Of note, our study is one of the few works that have done two separate determinations of these Ab. Regarding aPS/PT IgG Ab, positivity rates decreased from 32% to 28% for patients with APS when only considering those with persistent positive results. Unlike the study of Liu et al., which detected non-criteria aPLs in a considerable proportion of seronegative APS Chinese patients (23), none of the non-APS patients and none of the Others group in our cohort had aPS/PT IgG persistently positive and only 7% (2/30) of SLE patients had persistent positive results. This finding underlines the high association of aPS/PT IgG antibodies as biomarker for APS diagnosis. Since we did not include healthy donors, we could not analyze the clinical performance of this biomarker. We only calculated sensitivity and specificity for APS (APS and non-APS) versus disease controls (SLE and Others), but we may speculate that if we added a healthy control group to our cohort, these parameters would be better. However, in our group IgG aPS/PT exhibited higher specificity (84%) than IgM aPS/PT (50%).

Furthermore, we demonstrated a strong association of IgG aPS/PT Ab with APS criteria, thrombosis, triple positivity, GAPSS, and LA. No significant association was observed with obstetric complications, maybe due to the small number of patients included with these clinical manifestations. This contrasts with the results reported by Zigon et al., where the prevalence of aPS/PT Ab in the group of patients with obstetric complications was 13%. Moreover, aPS/PT Ab were the only biomarker associated with early recurrent pregnancy loss, as well as with late pregnancy morbidity and prematurity (24).

In another study, aPS/PT Ab was identified as an independent risk factor for venous thrombosis and the association of IgG aPS/PT was significant for obstetric abnormalities (25). In our study, 20 out of the 27 patients with positive LA were being treated with VKA, seven of which were also positive for IgG aPS/PT Ab, and of these seven patients, six had triple aPL positivity. Owing to the association between IgG aPS/PT Ab and LA, our study would support switching the determination of LA to aPS/PT IgG Ab in those cases of patients in whom anticoagulant treatment may interfere with the result of LA.

In our cohort, two patients with non-APS (one with thrombosis and the other with pregnancy morbidity) presented positivity for aPS/PT IgG at least in one determination, representing a prevalence of 9%. One of these patients was also positive for aPS/PT IgM in both determinations. Since the prevalence of these aPS/PT Ab in seronegative APS patients is higher in the literature (23, 26), we believe that it would be of interest to increase the size of this subgroup of patients in our cohort to confirm the results of previous studies. This group of patients represents a real challenge for clinicians, and additional laboratory testing could be useful for improving decisions on patient management.

In addition, we had two SLE patients with persistently positive aPS/PT IgG Ab: one had persistent triple aPL positivity, and the other was seronegative for classical aPL. Both of them have no thrombosis.

Nevertheless, there are some limitations in this study that must be highlighted. The main one is the small sample size, especially the group of patients with pregnancy morbidity.

In conclusion, our results support aPS/PT Ab as a promising biomarker for APS. The aPS/PT Ab assays showed high diagnostic efficiency for APS. The aPS/PT IgG Ab offer significant diagnostic utility for APS and can be used as an additional marker of thrombotic risk and as a surrogate marker of LA. Although additional studies are needed, our results strongly support the recommendations to include aPS/PT Ab as a new laboratory criteria biomarker for the classification of APS, especially in cases where patients have received anticoagulant treatment, since aPS/PT Ab are not affected by anticoagulant therapy and may be a substitute for LA determination.

## Data Availability Statement

The raw data supporting the conclusions of this article will be made available by the authors, without undue reservation.

## Ethics Statement

The studies involving human participants were reviewed and approved by the Ethical Committee of Hospital Clínic Barcelona (HCB/2019/1046). The patients/participants provided their written informed consent to participate in this study.

## Author Contributions

ER, OV, GE, and SL-S conceived the project and designed the study. LR performed the laboratory experiments. NE, CB, ER, OV, SL-S, and GN analyzed the results and wrote the manuscript. All authors contributed to the article and approved the submitted version.

## Conflict of Interest

SL-S, GN, CB, and MM are employed at Werfen, which sells autoantibody assays.

The remaining authors declare that the research was conducted in the absence of any commercial or financial relationships that could be construed as a potential conflict of interest.

The editor AS has declared past collaborations with one of the authors RC.

## Publisher’s Note

All claims expressed in this article are solely those of the authors and do not necessarily represent those of their affiliated organizations, or those of the publisher, the editors and the reviewers. Any product that may be evaluated in this article, or claim that may be made by its manufacturer, is not guaranteed or endorsed by the publisher.
